# Effect of a School-Based Intervention on Nutritional Knowledge and Habits of Low-Socioeconomic School Children in Israel: A Cluster-Randomized Controlled Trial

**DOI:** 10.3390/nu8040234

**Published:** 2016-04-21

**Authors:** Vered Kaufman-Shriqui, Drora Fraser, Michael Friger, Dikla Geva, Natalya Bilenko, Hillel Vardi, Naama Elhadad, Karen Mor, Zvi Feine, Danit R. Shahar

**Affiliations:** 1 Department of Public Health, Faculty of Health Sciences, Ben-Gurion University of the Negev, POB 653, Beer-Sheva 84105, Israel; veredk@ekmd.huji.ac.il (V.K.-S.); fdrora@bgu.ac.il (D.F.); friger@bgu.ac.il (M.F.); diklah@integristat.com (D.G.); natalya@bgu.ac.il (N.B.); hilel@bgu.ac.il (H.V.); 2 Braun School of Public Health , and Community Medicine, the Hebrew University Hadassah, Jerusalem 91120, Israel; 3 Centre for Research on Inner City Health, St. Michael’s Hospital, Toronto, ON M5B 1W8, Canada; 4 Ashalim: The Association for Planning & Development of Services for Children and Youth at Risk and Their Families, American Jewish Joint Distribution Committee, POB 3489, Jerusalem 91034, Israel; namanat@gmail.com (N.E.); karenm@jdc.org.il (K.M.); zvif@jdc.org.il (Z.F.)

**Keywords:** nutrition, low socioeconomic, childhood obesity, health promotion, pediatric nutrition, preschool children, school-based intervention

## Abstract

Early social and economic deprivation, associated with poor nutrition and physical inactivity, may lead to adverse health trajectories. A cluster-randomized controlled-trial examining the effect of a school-based comprehensive intervention on nutrition knowledge, eating habits, and behaviors among low socioeconomic status (LSES) school-aged children was performed. LSES school-aged children (4–7 years) and their mothers were recruited from 11 schools, located in one town. The intervention was implemented on three levels: children, mothers, and teachers. The intervention (IArm) included nutrition classes for children, mothers, and teachers and physical activity (PA) classes for children; the control (CArm) received PA only. Interventions were conducted by professional personnel, who were trained during in a two-day session to deliver the specific program in schools. Family data were obtained by parental interviews. Food knowledge observations, packed lunch records, and anthropometric measurements were obtained in school at baseline, six months, and at the end of the school year. Of 258 children enrolled, 220 (87.6%) completed the six-month program. Only children in the IArm improved their nutrition knowledge and eating-habits and increased food variety and fruit and vegetable consumption, quality score of packed lunches (*p* < 0.001 for all), habitual water drinking increased (*p* = 0.02), and decreased sweet-drink consumption (*p* = 0.05). A school-based comprehensive nutrition intervention targeting LSES population improved eating habits, nutritional knowledge, and healthier packed lunches.

## 1. Introduction

The double burdens of obesity and malnutrition are challenging public health issues worldwide, particularly among low socioeconomic status (LSES) children [1,2]. Diets rich in low-nutrient, energy-dense foods combined with sedentary lifestyles result in nutritional deficiencies and concurrent excessive weight gain relative to height increase [3,4]. Poor nutrition in childhood, in turn, is associated with adverse life-course health, chronic diseases, and lower cognitive performances [5,6]. In young children, a diet high in energy-dense foods leaves little room for nutrient-dense foods, which are crucial for optimal growth and development [7]. Teaching children about healthy nutrition when they are young, therefore, may help prevent the adverse outcomes described above. In addition, the evidence in the literature suggests that diet quality is a more important determinant in the prevention of obesity than is caloric intake [8,9].

Mothers play an important role in the development of children’s dietary habits by controlling availability, accessibility, and exposure to food [10,11] in the family. Mothers’ and teachers’ knowledge and role-modeling of a healthy lifestyle were shown to be associated with activity and healthier food choices of children [12,13,14]. In addition, nutritional education programs in school settings were shown to concomitantly increase the children’s knowledge and decrease the risk for an obesogenic environment in the family [15,16]. These findings support common knowledge that health habits and behaviors are established during early childhood and track into adulthood. These include food choice patterns, [17] levels of physical activity, and sedentary behaviors [18]. Although school is a critical period in human development, comprehensive interventions are scarce among children ages five and below who attend school classes, [19] even more so among LSES children [20]. Among the interventions conducted in school settings, some focused on educational components only [21,22] while others examined physical activity (PA) components exclusively; [23,24] only a few were comprehensive [25,26]. Most of the interventions showed limited success [14].

Our study examined a comprehensive school-based intervention focused on healthy lifestyle among the understudied population of LSES school children aged four to seven years during a single school year (overall 10 months). Following the ecological model [12] for promoting children’s healthy eating, the intervention was implemented among three domains that are considered as influencing children’s eating behaviors; (1) the individual level—the children; (2) the familial level—mothers; and (3) the educational level, represented by the school teachers.

Our intervention was designed to enhance children’s nutritional knowledge and to promote their mother’s adoption of healthy feeding habits, while considering food affordability constraints. Our primary objective was to improve the children’s nutritional habits; the secondary objective was to increase participants’ nutritional knowledge and levels of physical activity.

## 2. Materials and Methods

### 2.1. Study Population and Design

The cluster-randomized trial was conducted during a single school year in Beer Sheva, a big metropolis in southern Israel. Participants were enrolled during September to November 2008, and the intervention took place over the next three months, followed by a three-month follow up with the trial concluding in June 2009. Eligibility criteria were healthy children, aged 4–7, who were attending LSES school classes to which admission is determined based on household SES according to the local municipality criteria. Exclusion criteria included any chronic disease, developmental problems, attending a weight-control program, or any psychiatric problem of the child or a parent. Schools were eligible as long as they were not taking part in a similar program. Participants were recruited from the local municipality list of LSES schools enrolling 28–35 children per class. School classes in Israel for those ages are equal to junior and senior kindergarten classes; within one school class ages can range from as young as four years to as old as seven years, although the majority of children will be in the age range of five to six years. Each school in this city had one class. In order to minimize contamination, control schools were located in different neighborhoods of the city, and their teachers were not invited to attend teacher’s classes. The study received clinical trial registration: Unique Identifier: NCT01071551.

### 2.2. Sample Size

On the basis of data from an intervention conducted among similar populations, by the Association for Planning & Development of Services for Children and Youth at Risk and their Families, American Jewish Joint Distribution Committee (ASHALIM-JDC) which we received by personal communication, we calculated the sample size based on the assumption that healthy packed lunch score will increase by 30%. Using the software PS: Power and Sample Size Calculation software (Version 3.04.43; http://goo.gl/vC5uhf). We used *α* < 0.05 and *β* = 0.2, and a ratio of 3:1 between intervention arm (IArm) and control arm (CArm). An additional 30% was added to the sample size to account for dropouts and incomplete data. Using these criteria we enrolled in a 3:1 ratio of intervention to control, 180:60 families, giving overall 240 participants in the age range of four to seven years.

### 2.3. Randomization

After obtaining permissions from the local authorities, the Ministry of Education, and the Ethics Committee of Soroka University Medical Center (number 4712), a list of 16 schools meeting the eligibility criteria was prepared and the schools were numbered. The head of the Department of Education in the city randomly selected 11 numbers from a container for the IArm group and the remaining five schools were assigned to the CArm. The secretary then matched the numbers with the names of the schools. The randomization procedure was conducted in the presence of stakeholders and the study investigators. After randomization, all school attendees were recruited within schools that were approached sequentially. Letters were sent to families inviting them to meetings with school teachers and investigators to explain the study in detail. Mothers who agreed to participate signed an informed consent form and provided their contact details. All children participated in class activities, but information was collected only for children whose parents provided a written consent. The required sample size was achieved after recruiting four schools for the CArm and seven schools for the IArm.

### 2.4. Intervention Design

Following the ecological model for promoting children’s healthy eating [12], the intervention was implemented among teachers, mothers, and children. The IArm received the full program, which included nutrition-related intervention and PA while the CArm received PA lessons only. The intervention, which lasted three months, was developed by JDC-Ashalim, the development engaged all strata of stakeholders. All the lessons were delivered by professional personnel. Structured lessons to the parents were delivered by clinical dietitians, and economists specializing in family budget planning. Physical activity sessions were delivered by physical activity teachers.

The program (Appendix A) emphasizes healthy affordable nutrition using traditional and ethnically accepted diverse nutritional recommendations. Briefly, the main themes were increasing the consumption of fruits, vegetables, and legumes; decreasing the consumption of high fat and high sugar foods; decreasing sweet beverages and promoting water drinking instead. In addition, it promoted choosing healthy snacks, preparing healthy packed lunches, choosing a healthy breakfast, preparing nutritious low-budget meals, and increasing leisure time physical activity. Mothers and children in the intervention group received structured lessons in those themes. All children in the intervention group participated in 10 weekly nutrition sessions delivered by a clinical dietitian. Each lesson was 45 min long, using short lectures, stories, games, and songs to cover each topic. Parents received a weekly newsletter that paralleled the information offered to the children the same week. During this 15-week period, the intervention mothers participated in three meetings, two meetings were focused on preparing nutritious meals in low budget conditions, and a meeting for children and mothers focused on the preparation of healthy breakfasts and packed lunches.

IArm teachers received specific training to enhance the intervention effects (e.g., reinforcement of key messages) and health leadership training. Teachers were trained in various ways to compliment children who brought and ate healthy packed lunches. It was impossible to omit PA from the curriculum of the control group since PA classes, is the usual care in Israeli schools. All children received an identical curriculum of 15 weekly sessions of 45 min each of PA provided by professionals.

### 2.5. Data Collection

Data collection was performed by specially trained research assistants who were blinded to the intervention assignment. Mothers were interviewed regarding possible risk factors for childhood obesity. New immigrants from Ethiopia and the former Soviet Union were interviewed in the language of origin. Interviews were performed at baseline, at intervention termination (three months from baseline), and toward the end of the school year (six months from baseline). Interviews were structured, and included only closed ended questions. The assessors were blinded to group allocation.

### 2.6. Measures

#### 2.6.1. Demographics

Information on maternal age, parity, marital status, educational level, parental smoking status, and household SES were obtained during parental interviews. The family’s poverty status was defined by comparing the reported net household income per household resident to the national poverty line for families of similar size [27]. Countries of origin were grouped as follows: Israel; Europe and former USSR; Ethiopia and East Africa; and others. Maternal acculturation (the time from immigration to Israel to the interview) was divided into two categories: 0–9 years and 10+ years [28].

#### 2.6.2. Main Outcome Measures

##### Nutritional Habits

Nutritional habits were obtained from maternal interviews. Questions were related to the variety of foods consumed, and the consumption of vegetables, sweets and candies, sugar-sweetened beverage, and water. The questions were frequency based, and were taken from a food frequency questionnaire [29].

##### Packed Lunch Score

As the typical Israeli school day runs from 7:30 a.m. to 2:00 p.m., children usually eat breakfast at home, packed lunches are eaten at school between 10 and 11 a.m., and then larger, hot lunches are eaten later at home. The contents of the packed lunches were evaluated without prior notification every two weeks on random days of the week and on non-intervention days to maintain blinding.

Packed lunch contents were divided into five categories: sandwiches, snacks, sweetened drinks, fruit, and vegetables following the Choices Food Profiling criteria that determine whether foods are considered a healthy option within a defined category, according to their main nutrients [30]. Food items in each category were divided into high dietary quality and low dietary quality, and received scores of 1 and 0, respectively. The score for sandwiches was obtained by rating each of the three main ingredients separately, and then computing an average score: (1) Bread type (0 = white bread, 1 = whole wheat or rye); (2) Spread (0 = sweet or high fat spreads, e.g., jelly, 1 = unsaturated fat or vegetarian spreads, e.g., avocado, humus); (3) Filling (0 = foods containing saturated fat or high in sodium, e.g., salami, 1 = low-fat cheese, lean meat, and fish). All high-sugar, high-fat, and high-sodium snacks received 0 whereas low sugar breakfast cereals or low-fat yogurts received 1. All sugar-sweetened beverages received 0 and water or milk received 1. Any fruit or vegetable received the value 1. If a student carried more than one food item in a category an average grade was assigned, whereas a missing food category received zero. The arithmetic sum of the points given for each food category was used as a score of packed lunch quality; scores ranged from 0 to 5.

##### Physical Activity and Sedentary Behaviors

Leisure time PA of children was determined using the activity questionnaire from the Family Eating and Activity Habits Questionnaire (FEAHQ) [31]. Average daily screen time, *i.e.*, the time spent by the children watching television, videos, DVDs, or playing computer games (h/week), was calculated. Usual nighttime sleeping hours were reported in the interview.

### 2.7. Secondary Outcome Measures

#### 2.7.1. Evaluation of Children’s Nutritional Knowledge

Children’s nutritional knowledge was evaluated based on a questionnaire developed by Calfas *et al.* [32]. Briefly, the evaluation was performed in three stages using a progressive photo-test. First, children were presented with 19 photographs of light suppers, sandwiches, and main cooked meals, all of which depicted highly prevalent, local food items. Six options were healthy meals, 13 options were non-healthy meals, and the children were asked to define the meals as healthy or not. The second task was to choose the “healthier” meal from a pair of photographs. The third task was to grade four food photographs from the most unhealthy to the most healthy. This type of visual instrument was previously found to be appropriate for this age group in the United States and in the local population of school children in Israel [16,32]. A score (0 to 34) representing evaluation of the children’s nutritional knowledge was then calculated.

#### 2.7.2. Anthropometric Measurements

Mothers’ and children’s heights and weights were obtained in the schools using standard protocols. Children’s weights were measured with a portable digital scale (Tanita HD-318; Tanita Ltd., Arlington Heights, IL 60005, USA) while barefoot and dressed in light clothing. Height was measured with a portable stadiometer (SECA-217; Seca Ltd., Hamburg, Germany). The means of two weight and height measurements were used in the analysis. Body mass index (BMI) was calculated as weight in kilograms divided by height in meters squared (weight [kg]/height [m^2^]). We used the growth curves of the World Health Organization (WHO) to calculate BMI *z*-score values according to children’s age (months) and gender [33]. The World Health Organization (WHO) software AnthroPlus (Version V1.0.2; World Health Organization, Geneva, Switzerland) was used to obtain age-and sex-specific Body Mass Index (BMI—weight in kilograms divided by the square of the child’s height in meters) *z*-scores for each child.

### 2.8. Statistical Analysis

To compare prevalence in categorical variables, we used the χ^2^ or Fisher’s exact test. Means were compared using Student’s *t*-test. Change from baseline was calculated as percent of change from baseline and its significance was estimated using Wilcoxon rank sum test. Generalized estimating equation (GEE) model was used to analyze mean changes in outcomes over time. [34] The GEE method extends standard regression analysis to also account for the correlation between clustered measurements. Three longitudinal models were used to analyze changes in outcome measures between different time-points, using identity link and exchangeable working correlation matrix. The first model was fitted to the packed lunch score, the second to the nutritional knowledge score, and the third to the BMI *z*-scores. In all three models, the control group was the reference group and outcome measures were continuous. The explanatory variables in the models were intervention duration (time from baseline in months), and intervention group, models were adjusted to school cluster. The BMI model was also adjusted for baseline BMI-*z*-score, and included screen time (h/day) as an explanatory variable. Age and sex were not included in the analyses because BMI *z*-scores were computed relative to age- and sex-specific reference growth curves. We used multiple imputation (MI) to fill in for missing data for key outcome and exposure variables. For regression analyses, parameter estimates were based on the 10 imputation models were pooled together. *p* values < 0.05 were considered statistically significant, 95% Confidence Interval was calculated using the Wald Test. All the statistical analyses were performed using SPSS 18.0 for Windows (PASW Inc., Chicago, IL, USA).

## 3. Results

### 3.1. Characteristics and Adherence

**Figure 1:**
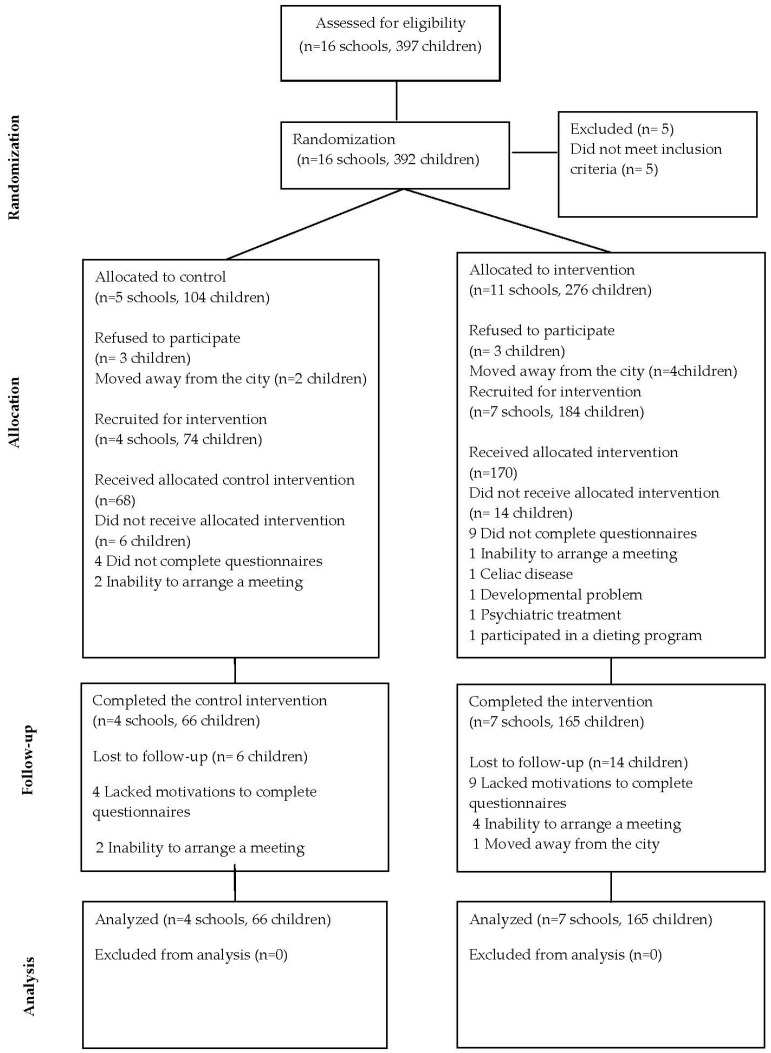
Flowchart of participants over the course of the study.

Of the 397 children attending the chosen schools, 5 did not meet the inclusion criteria; of the 392 randomized 6 refused to participate (3 in the IArm, and 3 in the CArm), and 6 moved away from the city (4 in the IArm, and 2 in the CArm); from the remaining 380 children (276 from 11 schools allocated for IArm, and 104 children from 5 schools allocated for the CArm), we sequentially recruited schools for the research (Figure 1). Parental consent to participate was obtained for 258 children. No difference was found between responders and non-responders in age, sex, and SES. Baseline data were obtained for 238 children, 231 children completed the three-month intervention, and 220 continued until the follow-up period. The overall adherence rates for completion of the program were recorded during each meeting. In the IArm 90.5% of the children attended all nutrition classes and 92% attended all PA classes. In the CArm 91% of the children attended all PA classes. Total adherence rates for the study were 94.1% and 88.2% in the IArm and in the CArm, respectively (Figure 1). Maternal adherence to parental meetings was 84%, teachers’ adherence to the training program was 90%. Baseline characteristics of participants are shown in Table 1. Arms were similar in most variables except for baseline BMI, BMI *z*-score, and mean screen time. Overweight and obesity were found in 71 (29.8%) children [34]. Mothers in the I Arm were older and more physically active.

### 3.2. Children’s Eating Habits

Changes in children’s habitual nutritional habits are presented in Table 2. Significantly greater increases were shown in the intervention than in the control arm in food variety, fruit and vegetable consumption, and habitual water drinking, as well as a marked decrease in consumption of sugar-sweetened beverages (all *p* < 0.05).

### 3.3. Quality of Packed Lunch

**Figure 2:**
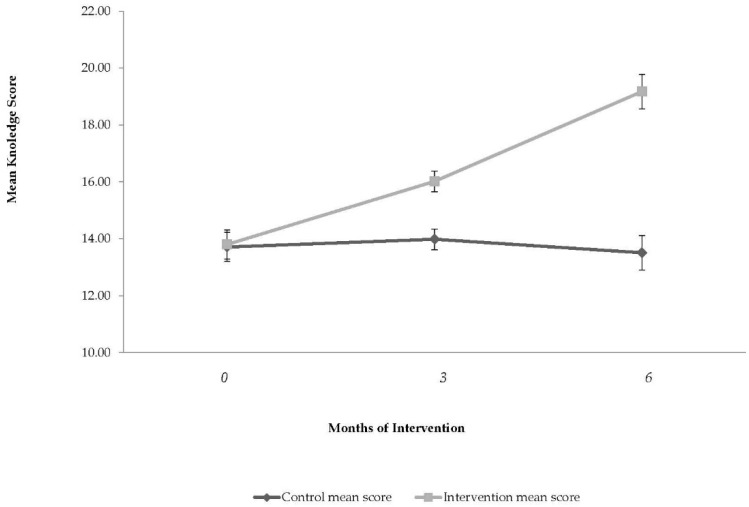
Changes in mean score for children’s nutritional knowledge by study arm at three months and six months from baseline. Vertical bars indicate standard errors, *p*-value for intervention group, time, and interaction between time and group. To statistically evaluate the changes in children’s nutritional knowledge score over time, generalized estimating equations were used, with the control group as the reference group. The explanatory variables were: time from baseline, school cluster, and intervention group.

The overall packed lunch score improved significantly over time in the IArm (Figure 2) in comparison to the CArm (1.16 ± 0.16, 120% *vs.* 0.41 ± 0.18, 42%, respectively, *p* < 0.0001). The overall variance explained by the model was 64%; 7% of the variance in the model was accounted for by baseline BMI *z*-score, 2% of the variance by school area, 10% of the variance by time from baseline (months), 6% of the variance by intervention group, and 39% of the variance by the interaction between intervention group and months from baseline. The main contributors to the increase in quality score of the children’s packed lunches were decreases in non-healthy snacks and sweet beverages and increases in fruit and vegetables; no change was found in the quality of sandwiches. The ICC for percent packed lunch score change at 3, and 6 months was small/moderate clustering (ICCs of 0.05, and 0.11).

### 3.4. Physical Activity

The mean PA time decreased significantly from baseline in the control arm compared with the intervention arm (−0.42 ± 0.01 h (−18.4%), −0.21 ± 0.01 h (−8.4%), respectively, *p* = 0.03). Total screen time increased significantly from baseline in the control arm compared with the intervention arm (0.54 ± 0.02 (18%) *vs.* no change, respectively, *p* = 0.001). No significant change was detected in mean sleeping hours in either group (*p* = 0.27). Either physical activity or sedentary behaviors did not cluster ICC = 0.00.

### 3.5. Children’s Nutritional Knowledge

**Figure 3:**
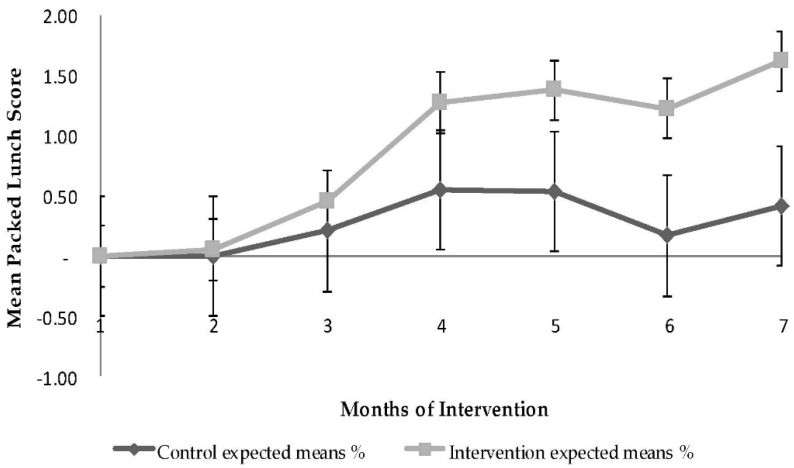
Changes in packed lunch score by study arm during a single school year. *p*-value was generated from the GEE model and represents *p*-values for intervention arm, time, and interaction between time and arm. Generalized estimating equations (GEE) were used to evaluate the changes in packed lunch score over time, with the control arm as the reference. Vertical bars indicate 95% CI.

Changes in the mean score for children’s nutritional knowledge are presented in Figure 3. The baseline knowledge score was 13.8 ± 0.5 in the control arm and 13.7 ± 0.6 in the intervention arm (NS). After three and six months of intervention, the mean score in the IArm increased by 2.2 ± 0.1 (16%) and 5.4 ± 0.1 (39%), respectively, compared with no change in the CArm (*p* < 0.001). The overall variance explained by the model was 49%, 9% of the variance in the model by baseline BMI *z*-score, 3% of the variance by school area, 7% of the variance by time from baseline (months), 4% of the variance by the intervention group, and 26% accounted for the interaction between intervention group and months from baseline. Change in nutritional knowledge score in three and six months did not cluster ICC = 0.00.

### 3.6. Weight

A significant reduction of 0.1 BMI *z*-score occurred in the entire study population (*p* = 0.003), in whom the BMI decreased from 16.3 ± 2.2 to 16.2 ± 2.4. The reduction was independently associated (in a GEE model) with a baseline BMI *z*-score (β = 0.88, *p* < 0.001), with time from baseline (months) (β = −0.21, *p* = 0.05), and packed lunch score (β = −0.07, *p* = 0.04), and an interaction between time from baseline (months) and screen-time (β = 0.06, 95% CI: −0.001–0.1, *p* = 0.05). Total variation explained by the model was 63%, baseline BMI *z*-score accounted for 20% of the variation, school area accounted for merely 0.15% of the variation, packed lunch score accounted for 15% of the variation, intervention group accounted for 2% of the variation, time accounted for 4% of the variation, screen time accounted for 3% of the variation, and the interaction between time from baseline (months) to screen time accounted for 18.85% of the variation. Change in BMI-z score in three and six months did not cluster ICC = 0.00.

## 4. Discussion

In this RCT among LSES school aged children (four to seven years) we found that the IArm increased nutritional knowledge and enhanced positive eating habits. These results were demonstrated by the higher consumption of water, fruits, and vegetables and in a greater food variety and lower consumption of sugar-sweetened beverages compared with the CArm. Children in the IArm whose mothers attended a nutrition education intervention focused on affordable, healthy food choices, and had significant improvements in the quality of their packed lunches. Schools represent an important dietary environment in which children can consume anywhere from one-third to two-thirds of their daily caloric intake if they are enrolled in a full-day program [35]. In this study, the packed lunches prepared at home and sent with the children to their schools reflected marked changes in parental feeding behaviors. Packed lunches were targeted by two previous interventions, both of which conducted short interventions with six-week follow ups and showed increases in terms of whole grains and vegetables consumed [36,37]. A recent Cochrane review focused on children age five years or younger, identified few randomized controlled trials investigating interventions to achieve the goal of increasing fruit and vegetable consumption found in our study [14], where only one school-based intervention succeeded in increasing fruit consumption [38]. In addition, examination of the components of the packed lunch score recorded in food observations showed a decrease in intake of low nutrition value snacks and sweet drinks. The decrease in sweet drink consumption was also reported during maternal interviews. The six-month follow up showed not only that this improvement was sustained, but that it even increased in many cases. Those results suggest a benefit in enforcing school policies on the consistency of packed lunches, and the benefit of school based education programs in influencing parental feeding behaviors.

The consumption of sugar-sweetened beverages is associated with higher BMI *z*-scores among young school aged children [39]. The majority of school-based interventions among school children have reported a decrease in the consumption of sugar-sweetened beverages [21,22,23,24,25,26]. In the Munch and Move trial conducted in Australia among children at the mean age of 4.4 years, lunchbox audit showed that children in the intervention group significantly reduced sweetened drinks by 0.13 servings [39,40]. The increase in water drinking among our study population is very important, since drinking water is an effective way not only to ensure adequate hydration, but may also help reduce dietary energy density and assist in the management of body weight [41].

After adjusting for baseline BMI-z score which distributed unevenly due to cluster randomization, both study arms showed moderate weight loss. We showed a reduction of 0.1 points in BMI *z*-score in both arms, and children in the intervention arm also stabilized their screen times. Very few comprehensive interventions have been performed among young school-aged children in general and among LSES populations in particular. Most of the interventions did not show significant effects on weight reduction [14,42]. The Romp & Chomp community intervention among preschoolers (two to five years old) showed a reduction of 0.7 percentage points in BMI *z*-scores compared with baseline values [26] while the first phase of the Hip-Hop to Health Jr. intervention in a minority population was effective in reducing subsequent increases in BMI in minority preschool children [43]. We believe that since both study arms received a professional PA program, their short-term success in weight loss may be due to that component of the intervention. The IArm stabilized screen time, which is a stronger predictor for increased daily energy intake than hours of physical activity, [44] may suggest a future impact on the prevention of childhood obesity [45]. Those findings reinforce the need for parental guidance on the association between children’s physical activity and sedentary activity and body weight.

### Limitations and Strengths

The main limitation of this study is that measurements were obtained during one school year without a long-term evaluation of the lasting impact of the intervention. However, tracking the children for another year was difficult in practice since on leaving daycare, they attend several different primary schools. While the reporting of children’s nutritional habits may have been biased by the classes offered to the mothers, the reported changes, in our opinion, are supported by the independent findings of changes observed in the compositions of packed lunches.

We also limited ourselves to interviews and observations within the daycare centers, as additional measures would have greatly reduced participation. Thus, the intervention achieved high adherence rates during an entire school year among a population in which adherence rates are known to be very low. Although children’s knowledge evaluation was modified from a validated tool, it was not validated amongst the study population, packed lunch score was not validated as well.

It is important to highlight that in order to benefit from such a program in a different country or culture there is a need to adapt programs performed in one country to the other.

The strengths of the study include the one-phase design, its relatively large study size, and the high adherence rates, and the economically appropriate nutritional intervention.

## 5. Conclusions

The comprehensive, school-based intervention was shown to be effective in improving eating habits, improving packed lunch quality, and stabilizing screen time of LSES school children aged four to seven years throughout a school year. The significant improvement in the quality of food brought by the children from home to class, accompanied by changes in nutritional habits, constitutes an important achievement with potential life course health implications. The trial highlights the impact of involving mothers and teachers in health promotion activities. Our results show that even a few focused economically appropriate nutrition education meetings with mothers that are supported by an ongoing educational activity of teachers and health professionals, can result in sustainable change in families’ and children’s nutritional knowledge, behaviors, and habits. The study reinforces the fact that either regular physical activity classes or nutrition education program and physical activity classes as part of the school curriculum, even during a short intervention time (three months), contribute to maintaining healthy body weight of school children. Further research should focus in the sustainability of healthy packed lunches, and the association between packed lunches and children’s whole nutrition.

## Figures and Tables

**Table 1:** Selected baseline characteristics of the study participants by study arm (*n* = 238).

Variable Children Preschool Classes	All (*n* = 238) (*n* = 11)	Control (*n* = 68) (*n* = 4)	Intervention (*n* = 170) (*n* = 7)	*p* Value
Age, (mean ± SD), months	63.4 ± 6.5	62.6 ± 6.9	63.8 ± 6.4	0.12
Gender, (male/female)	113/125	35/33	78/92	0.48
Weight *z*-score, (mean ± SD), kg	0.3 ± 1.3	0.5 ± 1.6	0.2 ± 1.2	0.10
Height *z*-score, (mean ± SD), cm	−0.1 ± 1.1	−0.1 ± 1.1	−0.1 ± 1.0	0.89
BMI *z*-score, (mean ± SD)	0.5 ± 1.3	0.9 ± 1.6	0.4 ± 1.1	0.01 *
BMI, (mean ± SD)	16.3 ± 2.2	16.8 ± 2.8	16.0 ± 1.9	0.01 *
Physical activity, (mean ± SD), h/week	2.4 ± 1.2	2.3 ± 1.2	2.5 ± 1.2	0.32
Sedentary hours, (mean ± SD), h/day	2.6 ± 1.4	3.0 ± 1.7	2.4 ± 1.2	0.01 *
Sleeping hours, (mean ± SD), h/night	9.8 ± 1.3	10.0 ± 1.3	9.8 ± 1.3	0.26
**Family characteristics**				
Below poverty line (%)	32.2	26.9	34.3	0.28
Number of siblings (%)				
0–1	42.9	43.5	42.7	0.92
2	23.5	21.7	24.2	
3+	36.6	34.8	33.1	
Religiosity (%)				
Secular	33.1	34.8	32.4	0.74
Traditional	43.3	44.9	42.6	
Orthodox	23.7	20.3	25	
Current parental smoking (%)	58.2	61.2	57.0	0.56
Single parents (%)	22.6	21.4	23.0	0.86

* Significant values, *p* < 0.05.

**Table 2:** Changes in children’s nutritional habits * by study arm during a single school year.

Nutritional Habit	Intervention Group		*p* Value ^†^
	Intervention	Control	
***n***	170	68	
**Eating a variety of foods**			
Baseline *n* (%)	92 (54.1)	33 (48.5)	0.45
3 months from baseline *n* (%)	133 (80.6)	37 (56.1)	
3 months change from baseline (%)	48.9	15.6	<0.001 ^a^
6 months from baseline *n* (%)	127 (79.4)	34 (56.6)	
6 months change from baseline (%)	46.7	16.8	<0.001 ^a^
**Daily vegetables eating**			
Baseline *n* (%)	96 (56.5)	36 (52.9)	0.41
3 months from baseline *n* (%)	134 (81.2)	41 (62.1)	
3 months change from baseline (%)	43.7	17.4	0.001 ^a^
6 months from baseline *n* (%)	126 (78.8)	37 (61.7)	
6 months change from baseline (%)	39.4	16.5	0.001 ^a^
**Consume sweet and candies on a daily basis**			
Baseline *n* (%)	91 (53.5)	33 (48.5)	0.23
3 months from baseline *n* (%)	59 (35.8)	20 (30.3)	
3 months change from baseline (%)	−33.2	−37.5	0.08
6 months from baseline *n* (%)	49 (30.6)	20 (33.3)	
6 months change from baseline (%)	−42.8	−31.3	0.13
**Habitual water drinking**			
Baseline *n* (%)	74 (43.5)	27 (39.7)	0.80
3 months from baseline *n* (%)	107 (64.8)	33 (50.5)	
3 months change from baseline (%)	49.1	25.9	0.003 ^b^
6 months from baseline *n* (%)	100 (62.5)	31 (51.6)	
6 months change from baseline (%)	43.7	30.1	0.02
**Daily consumption of sugar-sweetened beverage**			
Baseline *n* (%)	79 (46.5)	34 (50.0)	0.70
3 months from baseline *n* (%)	45 (27.3)	24 (36.4)	
3 months change from baseline (%)	−41.3	−27.2	0.02 ^b^
6 months from baseline *n* (%)	50 (31.2)	25 (41.7)	
6 months change from baseline (%)	−32.8	−16.6	0.05 ^b^

* Change from baseline was calculated by subtracting the percent of change at 4 and 6 months from baseline, percent presented as percent of change. Numbers represent the number of children reported to have the habitual nutritional habit, *n* = sample size at the specific point of time (baseline, 3 months, 6 months). ^†^ *p*-values for percent of change present differences between study arms that were calculated using the Wilcoxon rank sum test. ^a^ *p* value < 0.001, ^b^ *p* value < 0.05.

## Abbreviations

The following abbreviations are used in this manuscript:

MDPI	Multidisciplinary Digital Publishing Institute
DOAJ	Directory of open access journals
TLA	Three letter acronym
LD	linear dichroism

## Appendix A: Intervention program

The development of the intervention engaged all strata of stakeholders: municipality representatives, a committee of stakeholders with representatives from the ministry of health, education, and the local municipality oversaw the implementation of the program. The full model focuses on LSES families emphasizing healthy diet, and physical activity. The model was focused in affordable healthy food choices.

**Table A1:** Children’s nutrition intervention program (full intervention model) **.

Week	Objective	Themes	Specific Methods
1	Understand how the body works and the importance of healthy food and drinks	The importance of healthy food and drinks and active lifestyle to our health	Food samples (healthy *vs.* non-healthy). A doll (that the dietitian uses for example themes)
2	Understand the importance of water to our body	Why do we need water?	A doll (that the dietitian uses for example themes)
		How do we know we drink enough water?	Water and juice
3	Fruit and vegetables	Vitamins and minerals in fruit and vegetables	Examples of various fruit and vegetables
		The importance of eating fruit and vegetables of five colors	Preparation of a vegetable salad in five colors
4	Smart choices when eating sweets	What is in the sweets group?	Preparing a carrot cake
		What is a portion which is reasonable for a child to eat?	Examples of sweets portions
		Celebrating holidays with healthy/yummy food	A doll (that the dietitian uses for example themes)
5	Understand the importance of eating good proteins for growth	What are proteins?	Food samples
		Why are proteins important for us?	A doll (that the dietitian uses for example themes)
6	Understand the importance of legumes for healthy food choices	Identifying legumes	Cooking lentil soup
			A doll (that the dietitian uses for example themes)
7	Fats and oils in our food		
8	Ability to be make smart food choices	How to help your family prepare a healthy meal	Making smart choices from pictures
			Preparing a fruit salad
9	How to choose a healthy sandwich	What is a healthy sandwich?	Preparing of sample sandwiches
			Choosing a sandwich from pictures
			Composing healthy sandwiches from food models
10	Understanding what is a healthy meal	What contains a healthy meal? Food alternatives	Preparing a healthy meal from food models
		What should my body get during a whole day?	A doll (that the dietitian uses for example themes)

** All the lessons were delivered by a clinical dietitian, each lesson was 45 min long, the topics were taught through short lectures, stories, games, and songs. Mothers received a weekly newsletter that paralleled the information offered to the children that same week.

**Table A2:** School Teachers’ Intervention * (full model only, 60 h overall).

Week	Objective	Themes
**1**	Introduction to health promotion	Health promotion. Health promoting schools
**2**	Enhance teachers’ motivation and health leadership	The teacher as a leader of health promoting school
**3**	Adopting health promotion strategies to developmental stages in the life of children	Psychological development of school children
**4**	Disease prevention	The association between adverse lifestyle habits and future diseases
**5**	To understand the importance of breakfast	Breakfast importance
**6**	Enhance schoolteacher leadership by supplying behavioral tools	Conflict management with mothers and children
**7**	How to aid developing a positive body image among children	The development of body image of children during the early years of life
**8**	Supply the teacher with budgetary knowledge on how to prepare healthy snacks, sandwiches, and meals with low budgets in a culturally diverse school	Budget limitations and healthy eating
**9**	Introduction to physical activity of young children	Physical activity skills in school aged children
**10**	To promote hygiene in the school	Hygiene in the school
**11**	Experiencing physical activities adapted to young children	Physical activity skills in school aged children
**12**	Create ordinary activities with the teacher to enhance healthy school environment	Every-day life activities to promote healthy school

* The development of the intervention engaged all strata of stakeholders: municipality representatives, a committee of stakeholders with representatives from the ministry of health, education, and the local municipality oversaw the implementation of the program. The full model focuses on LSES families emphasizing healthy diet, and physical activity. The model was focused in affordable healthy food choices. All the lessons were delivered by professional personal: Clinical psychologists, clinical dietitians, physical activity teachers, public health nurses, and economists specializing in family budget planning.

**Table A3:** Mothers’ intervention (full model only).

Meeting Number	Children/Mothers	Themes	Specific Methods
1	Mothers only	Health food budget management	Comparing food prices Choosing alternative food items while considering cultural aspects
2	Mothers only	Healthy food budget implementation	Budgeting a household meal Composing a low budget family menu
3	Children + mothers	Preparing healthy sandwiches with a reasonable budget	Preparing healthy sandwiches Preparing individual sandwich periodical tables with each family

The development of the intervention engaged all strata of stakeholders: municipality representatives, a committee of stakeholders with representatives from the ministry of health, education, and the local municipality oversaw the implementation of the program. The full model focuses on LSES families emphasizing healthy diet, and physical activity. The model was focused in affordable healthy food choices. All the lessons were delivered by professional personal: economists specializing in family budget planning and clinical dietitians.

### Physical Activity Intervention Themes (children’s intervention, both groups)

Overall 15 group sessions, 45 min each, which included various activities in the school class to strengthen the child’s abilities in four main domains: strength, coordination, eye-hand coordination, and flexibility.
